# Comparison of the Effects of Automated and Manual Record Keeping on Anesthetists’ Monitoring Performance: Randomized Controlled Simulation Study

**DOI:** 10.2196/16036

**Published:** 2020-06-16

**Authors:** Man-Kei Tse, Simon Y W Li, Tsz Hin Chiu, Chung Wai Lau, Ka Man Lam, Chun Pong Benny Cheng

**Affiliations:** 1 Department of Applied Psychology Lingnan University Hong Kong China (Hong Kong); 2 Department of Anaesthesia and Intensive Care Tuen Mun Hospital Hong Kong China (Hong Kong)

**Keywords:** anesthesia information management system, automated record keeping, vigilance, situation awareness, mental workload

## Abstract

**Background:**

Anesthesia information management systems (AIMSs) automatically import real-time vital signs from physiological monitors to anesthetic records, replacing part of anesthetists’ traditional manual record keeping. However, only a handful of studies have examined the effects of AIMSs on anesthetists’ monitoring performance.

**Objective:**

This study aimed to compare the effects of AIMS use and manual record keeping on anesthetists’ monitoring performance, using a full-scale high-fidelity simulation.

**Methods:**

This simulation study was a randomized controlled trial with a parallel group design that compared the effects of two record-keeping methods (AIMS vs manual) on anesthetists’ monitoring performance. Twenty anesthetists at a tertiary hospital in Hong Kong were randomly assigned to either the AIMS or manual condition, and they participated in a 45-minute scenario in a high-fidelity simulation environment. Participants took over a case involving general anesthesia for below-knee amputation surgery and performed record keeping. The three primary outcomes were participants’ (1) vigilance detection accuracy (%), (2) situation awareness accuracy (%), and (3) subjective mental workload (0-100).

**Results:**

With regard to the primary outcomes, there was no significant difference in participants’ vigilance detection accuracy (AIMS, 56.7% vs manual, 56.7%; *P*=.50), and subjective mental workload was significantly lower in the AIMS condition than in the manual condition (AIMS, 34.2 vs manual, 46.7; *P*=.02). However, the result for situation awareness accuracy was inconclusive as the study did not have enough power to detect a difference between the two conditions.

**Conclusions:**

Our findings suggest that it is promising for AIMS use to become a mainstay of anesthesia record keeping. AIMSs are effective in reducing anesthetists’ workload and improving the quality of their anesthetic record keeping, without compromising vigilance.

## Introduction

An anesthesia information management system (AIMS) is a computer-based system that automatically imports real-time vital signs from physiological monitors to replace traditional handwritten records [1] and is increasingly being adopted by hospitals [2]. Despite the increasing popularity of AIMSs, recent studies on AIMSs mainly addressed the completeness of anesthetic records [3,4] but not the other attributes that are central to anesthetists’ monitoring performance, such as situation awareness and mental workload. The purpose of this paper was to report a full-scale high-fidelity simulation that compared the effects of AIMS use and manual record keeping on anesthetists’ monitoring performance.

Vigilance is the ability to maintain sustained attention over a long period of monitoring [5]. The most recent studies examining the effect of automated record keeping on vigilance were conducted 20 years ago [6,7]. Those studies focused on visual vigilance, which was operationalized as the time taken by participants to detect visual stimuli, including simulated abnormal values on a patient monitor [6] and flashing of an alarm light [7]. Anesthetists’ vigilance was not affected when record keeping was carried out by machines or assistants [6].

Situation awareness refers to one’s mental representation of the status of a dynamically changing environment. Situation awareness is measured at the following three levels: perception (level 1), comprehension (level 2), and projection (level 3) [8]. Situation awareness is critical to the administration of anesthesia because anesthetists need to monitor and be aware of numerous patient physiological variables (perception), detect unstable conditions and intervene appropriately (comprehension), and anticipate the effects of the intervention (projection) [9]. Situation awareness affects and is affected by mental workload, which is characterized as a subjective experience of the level of attentional demands imposed by performing tasks [10]. Noel suggested that anesthetists might become less attentive to the details of anesthetic events and patients’ status when they do not have to scan patients’ vital signs and write them down, as required in manual charting [11].

An AIMS would change the role of anesthetists from active processers of information to passive recipients [12,13]. As a result, anesthetists might be less attentive to the operating room (OR) surroundings and their patients’ status during monitoring. However, an AIMS is expected to reduce anesthetists’ subjective mental workload. Our three hypotheses specify that when compared with anesthetists who use manual record keeping, anesthetists who use AIMSs would have lower vigilance detection accuracy (H1), would have lower situation awareness accuracy (H2), and would experience lower subjective mental workload (H3).

## Methods

### Study Design and Approval

A parallel group experimental design was employed in this study. Ethical approval was obtained from Tuen Mun Hospital (TMH) (NTWC/CREC/17065) and Lingnan University (EC-063/1617). Written informed consent was obtained from all participants in advance and their data were deidentified.

### Participants

Participants were recruited from among the members of the Anaesthesia and Intensive Care Unit, TMH between September 2017 and March 2018. Participants were eligible if they were resident trainees or specialists. Based on the limited availability of anesthetists, we included 10 participants in each of the two conditions (ie, AIMS and manual), with a total of 20 participants. To achieve simple randomization of group assignment, one experimenter (MKT) placed 10 red (representing the AIMS condition) and 10 green (representing the manual condition) stickers into an opaque envelope and then randomly drew a sticker to generate the allocation sequence. As soon as participants enrolled in the study, they were assigned to a condition according to the allocation sequence.

### Simulation Design

A full-scale high-fidelity simulation was carried out in an OR at TMH. A clinical scenario specific for this study was designed by three anesthetists (THC, CPC, and KML). The scenario was designed to simulate uneventful monitoring with few critical incidents at intervals [14]. The scenario was set during the intraoperative portion of an emergency amputation below the right knee with general endotracheal anesthesia. It lasted for 45 minutes and comprised the following three phases: (1) preincident, (2) incident, and (3) postincident. The pre- and postincident phases were relatively uneventful, but the incident phase included the following three clinically relevant events: tourniquet pain, tourniquet deflation, and bleeding. The patient vital signs and progression were designed by an anesthetist (THC) and verified by a consultant anesthetist (CPC). When participants entered the simulation, they were asked to take over a case from a senior anesthetist (THC), who was a confederate in the study.

Apart from the participant, the simulation involved seven people, each with a specific role as follows: (1) senior anesthetist (THC); (2) runner nurse (a registered nurse colleague at TMH); (3) surgeon (CWL); (4) scrub nurse (KML); (5) patient simulator operator (CPC); and (6) two experimenters (MKT and SYWL). The confederates and the patient simulator operator were clinicians from TMH. The two experimenters were researchers from Lingnan University.

**Figure 1:**
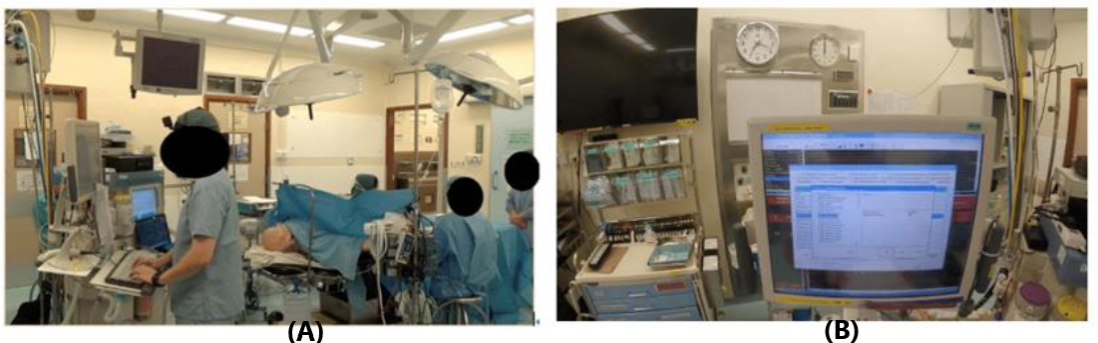
Video capture from the perspective of the operating room (A) and participant (B) while the participant was entering data into the anesthesia information management system during the simulation scenario.

Each simulation session was recorded by two digital video recorders; one captured a general view of the OR (Figure 1A) and the other was head-mounted (GoPro Hero 5; GoPro, San Mateo, California, USA) to capture the participant’s point of view (Figure 1B). A Fluke ProSim 8 Vital Signs Patient Monitor Simulator (Fluke Biomedical, Cleveland, Ohio, USA) was connected to a SimMan 3G (Laerdal Medical AS, Stavanger, Norway) patient simulator and a physiological monitor to display vital signs during the simulation.

Before the simulation began, participants were given a briefing to introduce them to the purpose of the study. The participants were then informed about the role of each confederate and the function of the patient simulator. In a training session, participants were given instructions and demonstrations on how to respond to assessments of vigilance, situation awareness, and mental workload during the simulation. Participants in the manual condition were also trained on how to manually complete an anesthetic record, because resident anesthetists at the hospital use an AIMS in their usual work practice. The simulation began when the senior anesthetist completed the handover to the participant. The participants were debriefed when the simulation was completed.

### Design of Situation Awareness Queries

The situation present assessment method (SPAM) [15] was used to measure participants’ situation awareness. At predetermined moments of the simulation, the experimenter MKT called the participants’ mobile phone to deliver situation awareness queries. The queries covered the three levels of situation awareness (perception, comprehension, and projection). For generating the situation awareness queries, we followed the process recommended by Endsley [16] to conduct a goal-directed task analysis (GDTA), which involved semistructured interviews, formulating a goal tree, and extracting and finally translating situation awareness requirements into scenario-specific queries. Details of the GDTA and situation awareness requirements are provided in Multimedia Appendix 1 and Multimedia Appendix 2, respectively. A total of nine situation awareness queries (Table 1) were generated with input from five anesthetists (CPC, KML, THC, an associate consultant, and a resident specialist).

**Table 1:** The nine situation awareness queries used in the scenario with their locations of information and their target answers.

Phase, Situation awareness queries		Location of the information	Target answer
**Preincident**			
	Level 1: What is the level of hemoglobin of the patient?	Preoperative assessment	Approximately 11
	Level 2: What is the most possible cause for the patient’s hypertension?	Physiological monitor (BP^a^, baseline BP) Understanding of the surgical procedure Medical knowledge	Tourniquet pain
	Level 3: If you do not provide any intervention, what would happen to the BP?	Physiological monitor (BP, baseline BP) Understanding of the surgical procedure Medical knowledge	Increase
**Incident**			
	Level 1: What is the patient’s baseline BP?	AIMS/manual record Physiological monitor	125/80
	Level 2: What is the most likely cause of the patient’s hypotension?	Physiological monitor (HR^b^, BP) Understanding of the surgical procedure Medical knowledge	Bleeding/volume loss
	Level 3: If you do not provide any intervention, what would happen to the end-tidal CO_2_?	Ventilator (CO_2_, baseline CO_2_, medical knowledge) Understanding of the surgical procedure	Increase
**Postincident**			
	Level 1: How much blood has the patient lost?	Suction bottle (volume of blood) Communication with nurses (volume of saline drip applied) Blood gauze	500-700 mL (within ±5% is acceptable)
	Level 2: Is the bleeding controlled? Why?	Suction tubing sound Suction bottle Physiological monitor (BP, HR) Surgical field (eg, blood gauze)	Yes, there is no more blood in suction tubing/HR and BP become normal
	Level 3: If you do not provide any intervention, what would happen to the hemoglobin level?	Medical knowledge Understanding of the surgical procedure Blood analysis	Increase. Not enough volume replacement, making the haemoglobin concentration higher. Or decrease. Due to severe blood loss

^a^BP: blood pressure.

^b^HR: heart rate.

### Primary Outcomes

**Figure 2:**
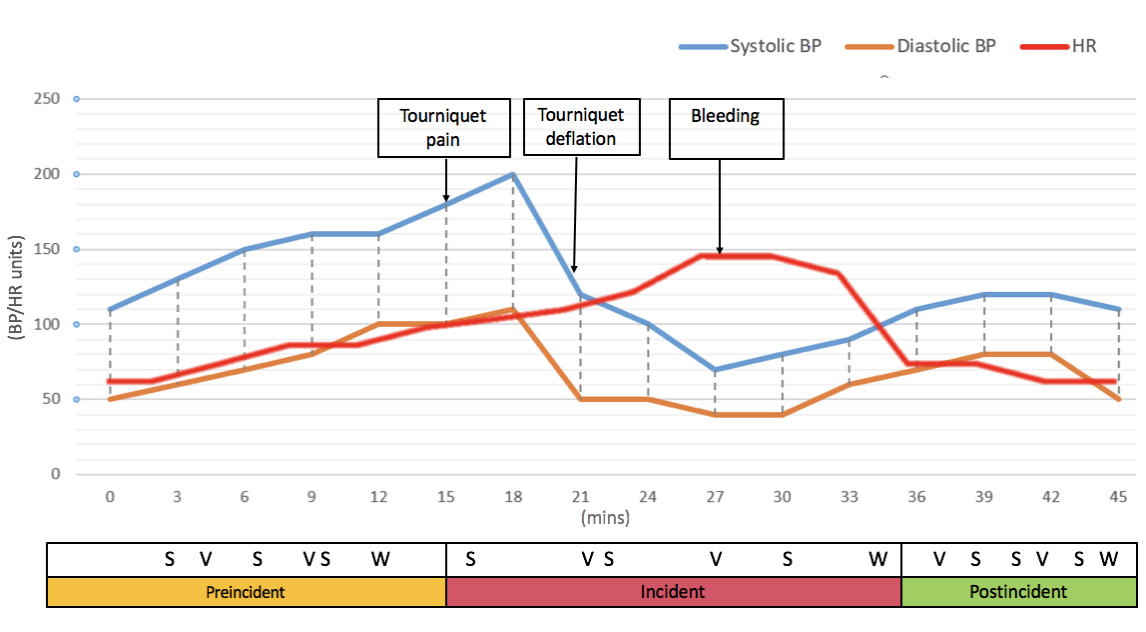
Design of the predetermined vital signs used in the clinical scenario of the simulation and the timeline of vigilance (V), situation awareness (S), and mental workload (W) assessments. BP: blood pressure; HR: heart rate.

There were three primary outcomes as follows: (1) accuracy of detecting suction tubing sounds (ie, vigilance detection accuracy), which were sounds made from actual suction tubing controlled by the scrub nurse (KML); (2) accuracy of correctly answering scenario-specific situation awareness queries (ie, situation awareness accuracy); and (3) self-reported mental workload ratings on The National Aeronautics and Space Administration Task Load Index (NASA-TLX) [17]. Measurements of the primary outcomes were performed by the experimenters MKT and SWL at predetermined times during the 45-minute scenario. Figure 2 shows how the measures were distributed over the three phases of the scenario.

### Secondary Outcomes

The secondary outcomes involved the distribution of the participants’ time across different task activities (ie, task time distribution), the quality of their anesthesia record (ie, anesthesia record completeness), and their attitude toward the AIMS. We assessed participants’ attitude toward the AIMS in terms of trust and acceptance, using a 45-item questionnaire (Multimedia Appendix 3) after the simulation was completed.

### Statistical Analysis

#### Operationalization of the Primary Outcomes

Vigilance was operationalized as detection accuracy for each participant. The score was calculated as the proportion (%) of the six tubing sounds that a participant detected. Situation awareness was operationalized as response accuracy, which was calculated as the proportion (%) of the nine situation awareness queries that the participant answered correctly. Each participant’s answers to the situation awareness queries were first evaluated against a predetermined marking scheme. When an answer did not match the target answer, an anesthetist (THC), who was blinded to the condition allocation, helped determine the accuracy of the answer according to expert judgement.

We performed the subjective mental workload measurement at the end of each simulation phase, in which participants rated each NASA-TLX dimension on a scale from 0 (lowest) to 100 (highest). The NASA-TLX comprises six dimensions (mental demand, physical demand, temporal demand, effort, frustration, and performance). The mean overall TLX score for each participant was calculated across the three simulation phases.

#### Operationalization of the Secondary Outcomes

Participants’ task activities in the simulation were video recorded and were reviewed to extract data on the different task activities. Task time distribution for each individual task category was computed as a percentage of the time spent on that category over the total time for all four tasks, including (1) entering record data, (2) monitoring the patient (eg, looking at the patient record, physiological monitor, anesthetic gas machine, or simulated patient), (3) performing patient care activities (eg, administering medication into patient’s intravenous access), and (4) interacting with the surgical team (eg, talking to the surgeon, asking the runner nurse to order medication, etc). Data were not coded for tasks that did not fall into any of the four task categories (eg, tidying up equipment wires, walking around the OR, etc).

Two raters assessed the participants’ anesthetic records for completeness using the 15-item checklist by Edwards et al [4], which was modified from the Australian and New Zealand College of Anesthetists’ recommendations on anesthetic records [18]. The two raters were an anesthetist (THC) and a consultant anesthetist (CPC), and they scored each checklist item with 1 (present), 0.5 (partially present), or 0 (absent) for the anesthetic records. The scoring was carried out by the raters independent from each other. The scores of individual checklist items were summed to produce a total score for each anesthetic record.

The trust and acceptance questionnaire had the following two parts: “trust in the AIMS” (adapted from a scale on trust in automated systems [19]) and “acceptance of the AIMS” (adapted from a scale based on the technology acceptance model [20-22]). All items in the questionnaire were rated on a 5-point Likert scale, with 1 indicating *strongly disagree*, 2 indicating *disagree*, 3 indicating *neutral*, 4 indicating *agree*, and 5 indicating *strongly agree*. Separate mean scores for trust and acceptance were calculated for each participant.

Prior to analysis, the Shapiro-Wilk test and Levene test were performed to assess the normality and homogeneity of variance, respectively, of the studentized residuals of the data. The independent sample *t* test was used to compare differences between the manual and AIMS conditions for normally distributed data. The Mann-Whitney *U* test was performed for non-normally distributed data.

According to the directions of the hypotheses, one-tailed significance tests were performed for the primary outcomes, whereas two-tailed tests were performed for the secondary outcomes. Task time distributions of the four tasks were compared between the two conditions with Bonferroni correction to obtain a more stringent alpha level of .0125 (.05/4).

## Results

### Response Rate

**Figure 3:**
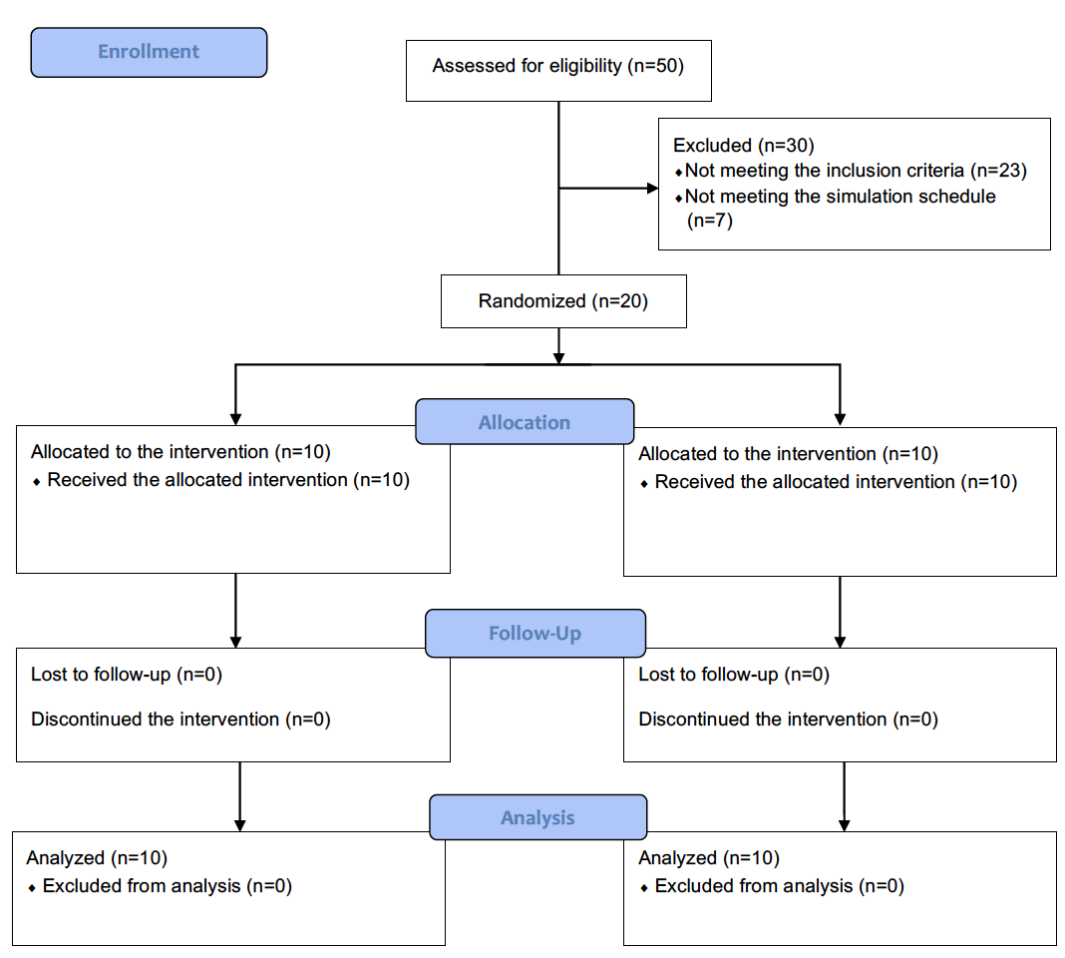
CONSORT disgram for the simulation study.

All 20 participants completed the trials without any dropout (Figure 3). Participants in the AIMS condition and those in the manual condition had comparable years of experience in anesthesia, with mean experience durations of 3.4 and 3.2 years, respectively.

### Primary Outcomes

There was no significant difference in vigilance accuracy between the AIMS (mean 56.7%, SD 32.6%) and manual conditions (mean 56.7%, SD 31.6%) (t_18_=0.00, *P*=.50, one-tailed); therefore, H1 was not supported. Although there was no significant difference in situation awareness accuracy between the AIMS (median 88.9%, range 66.7%-100%) and manual conditions (median 88.9%, range 77.8%-100%) (*U*=40.5, *P*=.48), we carried out a post-hoc power analysis using G*Power [23] on the basis of an emerging difference in trend between the two conditions. The achieved power (1 – β) calculated was 0.13, which was below the lowest conventionally acceptable level of 0.8. This suggests that the study did not have enough power to detect a difference in situation awareness accuracy between the AIMS and manual conditions. Therefore, H2 was inconclusive. However, we found that participants reported a significantly lower overall TLX score in the AIMS condition (mean 34.2, SD 12.5) than in the manual condition (mean 46.7, SD 11.5) (t_18_=−2.34, *P*=.02, one-tailed). Therefore, H3 was supported.

### Secondary Outcomes

Some video data were not coded (30% in the AIMS condition and 26% in the manual condition), as they either could not be classified or involved tasks that did not fall into our predefined task categories. Of the data that were coded according to the four task categories, only the proportion of time spent on record data entry differed significantly between the AIMS (mean 26.0%, SD 4.9%) and manual conditions (mean 33.7%, SD 6.9%) (t_18_=−2.87, *P*=.01, two-tailed). We also found that the level of completeness of anesthetic records was significantly higher in the AIMS condition (median 100%, range 93%-100%) than in the manual condition (median 75%, range 55%-87%) (*U*=0.000, *P*<.001, two-tailed). The two raters for record completeness had a high degree of reliability, with an average intraclass correlation coefficient of 0.893 and a 95% CI ranging from 0.68 to 0.96 (*F*_19,19_=11.59, *P*<.001). Finally, data from the trust and acceptance survey indicated that 45% (9/20) of respondents showed a positive attitude (*agree* or *highly agree*) of trust toward the AIMS and the remaining 55% (11/20) showed a neutral attitude. In terms of acceptance, 90% (18/20) of respondents showed a positive attitude (*agree* or *highly agree*) and 10% (2/20) showed a neutral attitude.

## Discussion

### Overall Findings

Despite the increasing adoption rate of AIMSs in hospitals [2], their effect on the monitoring performance of anesthetists has not been thoroughly examined. This study compared the effects of AIMS use and manual record keeping in terms of anesthetists’ levels of vigilance, situation awareness, and subjective mental workload with a randomized controlled trial in a high-fidelity simulation setting. The primary outcomes indicated that while there was no relevant difference in participants’ vigilance between AIMS use and manual record keeping, subjective mental workload was much lower among participants using the AIMS than among those using the manual method. However, the effect on situation awareness accuracy was inconclusive because the study was under-powered to detect its difference between the two conditions.

AIMS use might have two advantages over manual record keeping with respect to mental workload. First, the lower subjective mental workload with AIMS use might be a product of reduced physical movements. Informal inspection of our GoPro video data revealed that participants in the manual condition exhibited extensive head movements owing to the shifting of attention between the physiological monitor and the paper anesthesia chart. These movements may imply that more cognitive and perceptual activities (eg, remembering, looking, and searching for information) are involved in manual record keeping, and thereby, they result in higher subjective mental workload. Second, manual record keeping might have placed a high demand on participants’ prospective memory (remembering a future task) [24], because they needed to remind themselves to update vital signs on the paper chart regularly.

The secondary outcomes indicated further benefits of AIMS use. First, participants who used the AIMS spent about 8 percentage points less of their total time on record data entry than those who used manual record keeping. This result confirms previous findings that electronic record keeping allows anesthesia residents to spend less time on record keeping as compared to that with manual record keeping [7]. Second, AIMS use produced more complete anesthetic records than those produced by manual record keeping. This finding is consistent with the result of a previous study that retrospectively assessed 400 anesthetic records created by AIMS or manual record keeping methods [4] and reported more complete AIMS records than manual records. It is likely that AIMS use spares anesthetists from charting patients’ vital signs and allows them to spend more time on including other required information in the anesthetic records. Third, the attitude survey of AIMS use indicated that participants had a positive attitude toward trusting and accepting AIMS use in their practice.

Compared with previous studies on AIMS use that only examined visual vigilance [6,7], our study tested auditory vigilance. In this study, vigilance was operationalized as participants’ accuracy of detecting suction tubing sounds. This stimulus was chosen based on its clinical relevance, given that anesthetists often interpret it as a sign of patient blood loss during surgery. Although a direct comparison to visual vigilance might be impossible, our current results and those from previous studies suggest that AIMS use does not harmfully decrease anesthetists’ vigilance level [6,7]. However, irrespective of the type of record keeping, participants in this study demonstrated only a fair vigilance level in that they only detected, on average, 3.2 out of all 6 suction sounds (54%) in the vigilance assessments. We had not anticipated this result, but given the clinical importance of detecting suction sounds, this should be further investigated in future studies.

### Limitations

This study had six limitations. First, our simulated scenario only represented anesthetic cases that involve an uneventful period followed by critical incidents. Therefore, our findings can only be applied to the context of anesthesia with critical incidents. In anesthesia, many cases occur without any critical events. When the anesthetic procedure is uneventful, the effect of AIMS use on anesthetists’ vigilance and situation awareness might be different because complacency might arise, and this warrants further investigation.

Second, our participants were more accustomed to AIMS use than manual record keeping in their usual practice because junior anesthetists at TMH are trained on the AIMS but not on manual record keeping. Therefore, participants in our simulation had to be retrained on manual record keeping for comparison. While this retraining might seem artificial, it was the aim of TMH’s Department of Anaesthesia & ICU to investigate the tacit assumption of the effectiveness of AIMS use over manual record keeping. Retraining in the manual condition might have increased participants’ perceived mental workload, degraded their vigilance, and decreased their record keeping efficiency. This possible confounding factor could be addressed in future studies by sample screening or providing participants with prolonged training in manual record keeping.

Third, the findings of our study cannot be generalized to all models or brands of AIMSs. Other models of AIMSs might have different functions or interfaces and might interact with anesthetists differently.

Fourth, the participants, experimenters, and confederates were not blinded to the condition assigned to each participant owing to the nature of the manual and automated record keeping conditions.

Fifth, although our results suggest that AIMS use reduced the time spent on record data entry, it is unclear whether the time reduction led to an increase in time spent on monitoring patients or performing patient care activities. This could be addressed in future studies by examining how anesthetists reallocate the time saved with AIMS use to other tasks.

Sixth, we used a GoPro camera attached to each participant’s head in an attempt to capture visual data. However, the GoPro camera, at its best, could only provide us with the participant’s gaze direction. If accurate visual attention data are to be gathered, a mobile eye tracker should be used in future studies. Eye tracking data would allow for not only better inference of participants’ visual attention in general, but also identification of what activities they focus on when not interacting with the AIMS.

### Conclusions

Despite the increasing popularity of AIMSs in hospitals, no previous studies have analyzed their effects on comprehensive monitoring performance. The findings of this study provide support for the adoption of AIMSs in the OR by demonstrating a number of benefits of AIMS use, including reducing anesthetists’ perceived mental workload, saving their time spent on data entry, and producing complete anesthetic records, without compromising vigilance. Moreover, the majority of our anesthetists expressed a positive attitude toward trusting and accepting AIMSs in the OR.

The level of automation in health care is likely to increase as medical technology advances. It is important to know the effects that automation will have on patient care, as it could affect clinicians’ care quality and, ultimately, patients’ well-being and safety.

## Appendix

Multimedia Appendix 1 The goal-directed task analysis.

Multimedia Appendix 2 Pooled situation awareness requirements for the scenario.

Multimedia Appendix 3 Questionnaire for trust and acceptance of anesthesia clinical information systems.

Multimedia Appendix 4 CONSORT-eHEALTH checklist (V 1.6.1).

## Abbreviations

AIMS: anesthesia information management system
GDTA: goal-directed task analysis
NASA-TLX: National Aeronautics and Space Administration’s Task Load Index
OR: operating room
SPAM: situation present assessment method
TMH: Tuen Mun Hospital
