# Supplementary material for: Comparison of the Effects of Automated and Manual Record Keeping on Anesthetists’ Monitoring Performance: Randomized Controlled Simulation Study
Source: JMIR Hum Factors. 2020 Jun 16;7(2):e16036. doi: 10.2196/16036 (PMC7327599; doi:10.2196/16036)
Supplement: Multimedia Appendix 1 [file humanfactors_v7i2e16036_app1.pdf]

Main goal: Assure patient safety

Sub- goal 1. Deliver an effective general anaesthesia

1.1 achieve hypnosis/ sleep
- 1.1.1 Decide the anaesthesia depth
  - 1.1.1.1 Assess the patient past medical history/ pre-operative assessment
    - ❖ (Level 1) Record on heart- related diseases
    - ❖ (Level 1) Habit on drug abuse
- 1.1.2 Assess patients' response to hypnosis
  - ● *(decision) is the anaesthesia depth sufficient to the patient?*
    - ❖ (Level 1) current patient's heart rate

1.2 achieve analgesia/ pain relief
- 1.2.1 Assess patient's response to surgical procedure
  - ● *(decision) does the patient feel pain during surgery?*
    - ❖ (Level 1) current patient's heart rate

1.3 muscle relaxation

Sub –goal 2. Achieve the endpoints required for the given surgical procedure with the least risk to patients

2.1 Assess the operation risk
- ● (*decision) operation risk high or low?*
  - ❖ (Level1) Age of the patient
  - ❖ (Level 1) Past medical history of patient
- ● *(decision) are there multiple traumas/crush injury to other parts of the body?*
  - ❖ (Level 2)Location of traumas e.g lung
    - ✧ (Level 1) current patient's SpO2
  - ❖ (Level 2) Number of traumas
  - ❖ (Level2) Severity of the traumas
    - ✧ (Level 1) current patient's BP
    - ✧ (Level 1)Patient's baseline BP

2.2 Keep vital signs stable
- 2.2.1 Avoid excessive blood loss
  - 2.2.1.1 Assess blood loss
    - ● *(decision) Blood loss meets or exceed limit?*
      - ❖ (Level 3) projected blood loss
      - ❖ (Level 2) blood loss
        - ✧ (Level 1)Current BP
        - ✧ (Level 1)Current heart rate/ ECG
        - ✧ (Level 1)Current levels of hemoglobin
        - ✧ (Level 1)Ph value of blood
        - ✧ (Level 1)Sound of suction tubing
        - ✧ (Level 1) number of blood cloth
  - 2.2.1.2 Resolve excessive blood loss
    - ● *(Decision) how many changes are required?*

- ❖ (Level 3) number of changes
- ❖ (Level 3) type of intervention
- ❖ (Level 3) Projected impact on patient
- ❖ (Level 3)Volume of saline drip required

2.2.2 Avoid too low or too high blood pressure

2.2.2.1 Assess blood pressure

- *(decision) Blood pressure meets or exceeds limits?*
  - ❖ (Level 3) projected blood pressure
  - ❖ (Level 1)current blood pressure
  - ❖ (Level1) patient's baseline blood pressure

2.2.2.2 Resolve abnormal blood pressure

- *(Decision) how many changes are required?*
  - ❖ (Level 3) number of changes
  - ❖ (Level 3) type of intervention
  - ❖ (Level 3) Projected impact on patient
  - ❖ (Level 3) dosage of medication required

2.2.3 Avoid too low or too high heart rate

2.2.3.1 Assess heart rate

- *(decision) Heart rate meets or exceeds limits?*
  - ❖ (Level 3) projected heart rate
  - ❖ (Level 1)current heart rate
  - ❖ (Level 1)) shape of ECG
  - ❖ (Level 1) Patient's baseline heart rate

2.2.3.2 Resolve abnormal heart rate

- *(decision)How many changes are required?*
  - ❖ (Level 3) number of changes required
  - ❖ (Level 3) Projected intervention
  - ❖ (Level 3)Projected impact on patient

2.3 Avoid intra-operative complication

2.3.1. Assess the risk of heart attack during surgical procedure

- *(decision) risk is high, medium or low?*

  - ❖ (Level 3) Projected Patient's end tidal CO2 when tourniquet is released
  - ❖ (Level 3) Projected patient's level of electrolyte e.g. potassium in blood
  - ❖ (Level 1) current Patient's end tidal CO2 when tourniquet is released
  - ❖ (Level 1) current patient's level of electrolyte e.g. potassium in blood
  - ❖ (Level 1) shape of ECG

2.3.2. Assess the risk of acute renal failure during surgical procedure

- *(decision) risk is high, medium or low?*
    - ❖ (Level 3) current patient's level of electrolyte e.g. potassium in blood
    - ❖ (Level 1) current patient's level of electrolyte e.g. potassium in blood
    - ❖

2.4 Provide good ventilation

2.4.1 Assure stable end-tidal CO2

2.4.1.1 Assess end-tidal CO2

- ❖ (Level 3) projected end-tidal CO2
- ❖ (Level 1) current end-tidal CO2

2.4.1.2 Resolve unstable end-tidal CO2

2.5 Maintain patient's body temperature in a normal range

2.5.1 Assess body temperature

- ❖ (Level 1) current patient's body temperature

2.5.2 Resolve hypothermia

Sub-goal 3. Enable a better post-operative management

3.1 Relieve post-operation pain

3.2 Avoid post-operation complication

3.3 Decide a proper place for post-operative care
