# Supplementary material for: Comparison of the Effects of Automated and Manual Record Keeping on Anesthetists’ Monitoring Performance: Randomized Controlled Simulation Study
Source: JMIR Hum Factors. 2020 Jun 16;7(2):e16036. doi: 10.2196/16036 (PMC7327599; doi:10.2196/16036)
Supplement: Multimedia Appendix 2 [file humanfactors_v7i2e16036_app2.pdf]

| SA requirement | | |
|---|---|---|
| Level 1 | Level 2 | Level 3 |
| Patient's medical history<br>• record on heart- related diseases<br>• Habit on drug abuse<br>Patient's demographic information<br>• Age of the patient<br>Patient's vital signs<br>• heart rate<br>• SPo2 levels<br>• Bp<br>• baseline Bp<br>• baseline blood pressure<br>• baseline heart rate<br>• end tidal CO2<br>• body temperature<br>• shape of ECG<br>Patient's HemoCue result<br>• levels of hemoglobin<br>• PH value of blood<br>• level of electrolyte e.g. potassium in blood<br>Surgical field<br>• suction tubing sound | Operation risk<br>• surgical procedure involved<br>• Other possible traumas in patient's body apart from right feet<br>Blood loss<br>• Severity<br>• Volume<br>Patient's response to anaesthesia<br>• hypnosis/ sleep<br>• analgesia/ pain relief<br>• muscle relaxation | Projected impact of excessive blood loss on patient<br>• Increasing HR<br>• Decreasing blood pressure<br>• Decreasing hemoglobin<br>Projected impact on patient when tourniquet is released<br>• Increasing end-tidal CO2<br>• Increasing electrolyte in blood<br>• Decreasing blood pressure<br>Projected risk of intra-operative complication<br>• Heart attack<br>Projected intervention to unstable vital signs<br>● Number of changes required<br>● Impact on patient<br>● Type of intervention |
