# Supplementary material for: Comparison of the Effects of Automated and Manual Record Keeping on Anesthetists’ Monitoring Performance: Randomized Controlled Simulation Study
Source: JMIR Hum Factors. 2020 Jun 16;7(2):e16036. doi: 10.2196/16036 (PMC7327599; doi:10.2196/16036)
Supplement: Multimedia Appendix 3 [file humanfactors_v7i2e16036_app3.pdf]

“Trust and acceptance of ACIS” questionnaire

This questionnaire contains 45 questions related to your trust and acceptance in using Anaesthesia Clinical Information System (ACIS) in your clinical practice.

Instruction: Please respond to all statements in the questionnaire. ***“The system” used in the statements refers to ACIS.*** For each of the following statements, read it carefully and circle the number that best describes your level of agreement. *(1= strongly disagree, 5= strongly agree)*. Please note that there is no ‘right’ or “wrong” answer, just mark what is true for you. Please do not spend too much time on each statement, just answer based on your intuition.

Part I. Trust in ACIS

		Strongly disagree	Disagree	Neutral	Agree	Strongly agree
1.	I am suspicious of the system's intent, action, or outputs	1	2	3	4	5
2.	The system is deceptive	1	2	3	4	5
3.	The system has integrity	1	2	3	4	5
4.	The system is dependable	1	2	3	4	5
5.	The system behaves in an underhanded manner	1	2	3	4	5
6.	I am confident in the system.	1	2	3	4	5
7.	I can trust the system	1	2	3	4	5
8.	The system’s actions will have a harmful or injurious outcome	1	2	3	4	5
9.	I am familiar with the system.	1	2	3	4	5
10.	The system provides security	1	2	3	4	5
11.	The system is reliable.	1	2	3	4	5
12.	I am wary of the system.	1	2	3	4	5

Part II. User’s acceptance

13.	The use of this system may imply major changes in my clinical practice	1	2	3	4	5
14.	I have no problem with the quality of the system's output.	1	2	3	4	5
15.	Using the system in my job increases my productivity.	1	2	3	4	5
16.	I find the system to be useful in my job.	1	2	3	4	5
17.	I find it interesting to use this system for the monitoring of my patients.	1	2	3	4	5
18.	I think it is a good idea to use this system to monitor my patients.	1	2	3	4	5
19.	The quality of the output I get from the system is high.	1	2	3	4	5
20.	In my job, usage of the system is relevant.	1	2	3	4	5
21.	I would have difficulty explaining why using the system may or may not be beneficial.	1	2	3	4	5
22.	Using the system improves my performance in my job.	1	2	3	4	5
23.	The use of this system may promote good clinical practice	1	2	3	4	5
24.	My interaction with the system is clear and understandable.	1	2	3	4	5
25.	The use of this system is beneficial for the care of my patients.	1	2	3	4	5
26.	In my job, usage of the system is important.	1	2	3	4	5
27.	I find it easy to get the system to do what I want to do.	1	2	3	4	5
28.	Assuming I have access to the system, I intend to use it.	1	2	3	4	5
29.	The use of this system may interfere with the usual follow-up of my patients	1	2	3	4	5
30.	The use of this system is compatible with my work habits	1	2	3	4	5

31.	I find the system to be easy to use	1	2	3	4	5
32.	The results of using the system are apparent to me.	1	2	3	4	5
33.	I have no difficulty telling others about the results of using the system.	1	2	3	4	5
34.	Given that I have access to the system, I predict that I would use it.	1	2	3	4	5
35.	Interacting with the system does not require a lot of my mental effort.	1	2	3	4	5
36.	I believe I could communicate to others the consequences of using the system.	1	2	3	4	5
37.	In my opinion, the use of this system will have a positive impact.	1	2	3	4	5
38.	Using the system enhances my effectiveness in my job.	1	2	3	4	5

Part III. Demographic information

39. Gender: M / F
40. Age: ________
41. Hospital name: ______________________
42. Job title: _____________________
43. Years of experience in anaesthesia: __________
44. Years of experience using ACIS:____________
45. a) Did you receive any training in learning to use ACIS? Yes / No

    b) If yes, how long did you spend on learning to use ACIS?

    ____

The End. Thank you.
